# CD3^+^CD4^neg^CD8^neg^ (double negative) T lymphocytes and NKT cells as the main cytotoxic-related-CD107a^+^ cells in lesions of cutaneous leishmaniasis caused by *Leishmania* (*Viannia*) *braziliensis*

**DOI:** 10.1186/s13071-017-2152-2

**Published:** 2017-05-03

**Authors:** Raquel Ferraz, Clarissa F. Cunha, Maria Inês F. Pimentel, Marcelo R. Lyra, Tatiana Pereira-Da-Silva, Armando O. Schubach, Alda Maria Da-Cruz, Alvaro Luiz Bertho

**Affiliations:** 1 Laboratory of Immunoparasitology, Oswaldo Cruz Institute, FIOCRUZ, Rio de Janeiro, RJ Brazil; 2 Flow Cytometry Sorting Core Facility, Oswaldo Cruz Institute, FIOCRUZ, Rio de Janeiro, RJ Brazil; 3 Laboratory of Surveillance for Leishmaniasis, Evandro Chagas National Institute of Infectology, FIOCRUZ, Rio de Janeiro, RJ Brazil; 4 Laboratory of AIDS and Molecular Immunology, Oswaldo Cruz Institute, FIOCRUZ, Rio de Janeiro, RJ Brazil; 5 Laboratory of Interdisciplinary Medical Research, Oswaldo Cruz Institute, FIOCRUZ, Rio de Janeiro, RJ Brazil

**Keywords:** Flow cytometry, Cytotoxicity, CD107a, Double-negative T lymphocytes, NKT cells, Lesion, Human cutaneous leishmaniasis, *Leishmania* (*Viannia*) *braziliensis*

## Abstract

**Background:**

Cutaneous leishmaniasis (CL) is caused by *Leishmania* (*Viannia*) *braziliensis,* which infects dermal macrophages and dendritic cells, causing an intense immune-mediated-tissue inflammation and a skin ulcer with elevated borders that can heal spontaneously or after antimonial therapy. The resolution of lesions depends on an adaptive immune response, and cytotoxic cells seem to have a fundamental role in this process. The aim of this study is to better understand the role of cytotoxicity mediated mechanisms that occur during the immune response in the CL lesion milieu, considering distinct cytotoxic-related CD107a^+^ cells, such as CD8^+^, CD4^+^, CD4^neg^ CD8^neg^ (double-negative, DN) and CD4^+^CD8^+^ (double-positive, DP) T lymphocytes, as well as NK and NKT cells.

**Methods:**

Lesion derived cells were assessed for T cell subpopulations and NK cells, as well as CD107a expression by flow cytometry. In addition, cytometric bead array (CBA) was used to quantify cytokines and granzyme B concentrations in supernatants from macerated lesions.

**Results:**

Flow cytometry analyses revealed that NKT cells are the major CD107a-expressing cell population committed to cytotoxicity in CL lesion, although we also observed high frequencies of CD4^+^ and DN T cells expressing CD107a. Analysing the pool of CD107a^+^-cell populations, we found a higher distribution of DN T cells (44%), followed by approximately 25% of NKT cells. Interestingly, NK and CD8^+^ T cells represented only 3 and 4% of the total-CD107a^+^-cell pool, respectively.

**Conclusions:**

The cytotoxicity activity that occurs in the lesion milieu of CL patients seems to be dominated by DN T and NKT cells. These findings suggest the need for a reevaluation of the role of classical-cytotoxic NK and CD8^+^ T cells in the pathogenesis of CL, implicating an important role for other T cell subpopulations.

## Background

Leishmaniasis is a group of diseases caused by different species of protozoan parasites from the genus *Leishmania* and is a major neglected tropical disease affecting humans globally [1]. In Brazil, American tegumentary leishmaniasis (ATL) is caused mainly by *Leishmania* (*Viannia*) *braziliensis* and is present in all states, including Rio de Janeiro, where it is endemic. The disease presents a broad spectrum of clinical, immunological and histopathological manifestations, ranging from self-healing localised cutaneous leishmaniasis (CL) to destructive mucosal leishmaniasis (ML). CL is the most frequent clinical form of ATL and is characterised by the parasitic infection of derma, which results in an intense immune-mediated tissue inflammation and a skin ulcer with elevated borders that can heal spontaneously or after antimonial therapy. *Leishmania* induces a chronic granulomatous inflammatory disease, given it involves the recruitment of lymphocytes, plasmocytes and macrophages to the skin [2]. Several authors have demonstrated that the pathogenesis of ATL is dependent on the cellular immune response and it seems to affect the clinical outcome of the disease by T-lymphocyte effector functions and cytokine profiles [3–5]. Thus, even though the host immune response contributes to protection, it may also be deleterious favouring the establishment and persistence of the disease. Studying the cellular immune response in ATL lesions allows us to propose mechanism involved in the formation, persistence or healing of leishmaniasis lesions.

Although CD4^+^ T cells are clearly an important source of cytokines to activate leishmanicidal activities, it is equally evident that several other cell types are essential for an efficient immune response in the lesion microenvironment of leishmaniasis. In this context, some reports have shown that CD8^+^ T cells may have an imperative role in the immune response in this disease, mainly acting as IFN-γ producers, as well as cytotoxic cells. However, their role as a beneficial or deleterious subpopulation is controversial, depending on their functional status.

It is worthy to highlight that the majority of studies about the immune response in ATL were performed with samples obtained from peripheral blood of patients; however, the immunopathogenic events take place in situ, which highlights the importance of studying the lesion microenvironment. Previous observations from our group have shown an expansion of CD8^+^ T lymphocytes in the inflammatory infiltrate, suggesting that they are recruited to the site of infection, and therefore committed to the healing process of the CL lesion [6–12]. In contrast, other authors have associated CD8^+^ T lymphocytes with tissue injury in CL and ML [12–17]. Observing cell subpopulations in CL lesions, the cell infiltration and pathology suggest that tissue damage is a consequence of the immune response, mostly related to T-cell-mediated cytotoxicity, rather than the parasite itself [18]. Moreover, other authors have shown that the production of granzyme A is associated with lesion progression, while granzyme B is necessary for cytolysis of *L. braziliensis*-infected monocytes, but also induces tissue damage in CL patients [16, 17].

Aside from CD8^+^ T lymphocytes, other cell populations, like NK and NKT cells, are known as cytotoxic cells and have a crucial influence on the development of the disease or cure. Furthermore, other two T-lymphocyte populations, CD4^neg^CD8^neg^ double-negative (DN) and CD4^+^CD8^+^ double-positive (DP), can also contribute to the cytotoxic activity. Hence, the controversy about the cytotoxicity in the immunopathogenesis of ATL needs further study.

Cytotoxic T lymphocytes and NK cells share the same route of cytotoxicity, by exocytosis of lytic granules. This activity is performed by perforin and granzyme, which are stored in lipid bilayer vesicles, containing lysosomal-associated membrane protein (LAMP), including CD107a (LAMP-1). This vesicle fuses with the plasma membrane at the time of exocytosis, thus mobilising CD107a to the cell surface, indicating a functional degranulation and this phenomenon has been exploited in cytotoxicity studies [19–21].

Since lesions of CL patients are the hallmarks of the clinical course of the disease, it is fundamental to explore how the cytotoxic activity might be involved in the lesion environment. Some authors have previously reported a heterogeneity in the distribution of T-cell subsets in the lesion milieu [8, 9, 21], while other reports concerning in situ immunopathological analysis, have helped understand the local immune response, although the histopathological patterns may differ within the same lesion, depending on the site of ulcer or the duration of the disease [22–24]. Thus, it is imperative to determine which cell subset effectively prevails in the lesion environment and their cytotoxic profiles.

The results shown here were based on the investigation of the frequency and distribution of T lymphocyte subpopulations, NK and NKT cells in lesions of patients with their first manifestation of CL. Moreover, cell subpopulation cytotoxic activity through CD107a staining and granzyme B quantification was determined using flow cytometry. We also investigated correlations between the immunological findings and clinical features, to determine protective or inflammatory profiles, established at lesion milieu.

## Methods

### Subjects

Eighteen patients were selected according to the inclusion criteria of a clinical and epidemiological history compatible with CL. All of the 18 patients enrolled in the study were diagnosed for leishmaniasis and were known to have acquired the disease in *L. braziliensis*-endemic areas of Rio de Janeiro, Brazil [2]. All of them were recruited at Leishmaniasis Surveillance Laboratory, Evandro Chagas National Institute of Infectology (INI), Oswaldo Cruz Foundation (FIOCRUZ), Rio de Janeiro, Brazil. The main clinical features of the studied patients are described in Table 1. The following criteria were used for diagnosis: (i) positive Montenegro skin test (MST); (ii) direct detection of parasites by light microscopy observation; (iii) isolation of *Leishmania* parasites by culture fragment in Nicolle-Nevy-McNeal (NNN) medium; and histopathologic analysis of the inflammatory infiltrate. We maintained the fragments of lesion biopsy in PBS supplemented with antimicrobials (penicillin and streptomycin) for a maximum of 4 hours before processing. The species of isolated parasites were characterised by isoenzyme electrophoresis profiles [25]. All patients were submitted to meglumine antimoniate treatment according to the guidelines of the Brazilian Ministry of Health.

**Table 1:** Demographic and clinical information of patients included in the study

Number of volunteers	18
Sex: Male/Female	17/1
Age (years)^a^	39.5 ± 6.0
Number of lesions^a^	1.0 ± 0.4
Diameter of lesions (mm)^a^	41.3 ± 5.0
Montenegro Skin Test (MST) (mm)^a^	19 ± 2.8
Duration of disease (months)^a^	57.9 ± 15.0

^a^Mean ± Standard deviation

### Collection and processing of tissue sample

Incisional skin biopsy was performed for diagnosis purposes and experimental procedures. Cells were obtained from lesions as described elsewhere [7]. Briefly, after local anaesthesia with lidocaine, the biopsy was performed using a 6 mm punch including 1/3 edge of the lesion. The obtained fragment was immediately stored in RPMI 1640 (Gibco, Carlsbad, CA, USA), supplemented with HEPES (10 mM), L-glutamine (1.5 mM), 2-mercapto-ethanol (1 mM), penicillin (200 UI), and streptomycin (200 μg/ml) enriched with 20% of fetal calf serum (all reagents from Sigma-Aldrich Co (St Louis, MO, USA) for up to 4 hours at 4 °C. We placed the skin specimen onto a cell dissociation sieve (Tissue Grinder kit, Sigma-Aldrich), stripped off the subcutaneous fat, and macerated it on a 64 μm-stainless steel mesh in Petri dish, containing supplemented RPMI 1640 medium. After that, we transferred the cell suspension with macerated tissue to a 15 ml Falcon tube and let it rest for five minutes to decant large epithelial cells, dermal cells and fat tissue. Afterwards, we collected the cell-containing supernatant, centrifuged to pellet cells and separate the supernatant for cytometric bead array (CBA). To obtain a more pure-cell sample, we resuspended the cell pellet and filtered the cell suspension into Falcon® 12 × 75 mm tube with cell strainer cap with 35 μm nylon mesh (BD Biosciences, San Jose, USA). Additionally, we resuspended the cells in PBS supplemented with 0.1% sodium azide and 2% fetal calf serum (PBSaz, Sigma-Aldrich) and adjusted to 1 × 10^6^ cells per 50 μl, for 6-color flow cytometry staining, described below.

### Flow cytometry

Flow cytometry staining protocol was performed as previously described [11]. Briefly, after collection and processing of tissue specimen*,* cells were stained for surface markers with a panel of monoclonal antibodies (MAbs), as follow: anti-CD3; anti-CD56; anti-CD8 (Beckman Coulter, Kendon, FL, USA); anti-CD4 and anti-CD107a (BD, Bioscience, San Jose, CA, USA). The cells were also stained with 7-aminoactinomycin D (7-AAD; Beckman Coulter) for cell viability determination. After 20 min on ice and protected from light, we washed cells once and incubated with BD FACS lysing solution (BD Biosciences) to lyse erythrocytes.

In each sample, 500,000 events were acquired using MoFlo Astrios Cell Sorter Flow Cytometer (Beckman Coulter) in a low-flow rate (max 800 eps) to avoid clogging. Single stained controls were used to set compensation parameters, as well as fluorescence-minus-one (FMO) and isotype controls were used to set analysis gates.

**Fig. 1:**
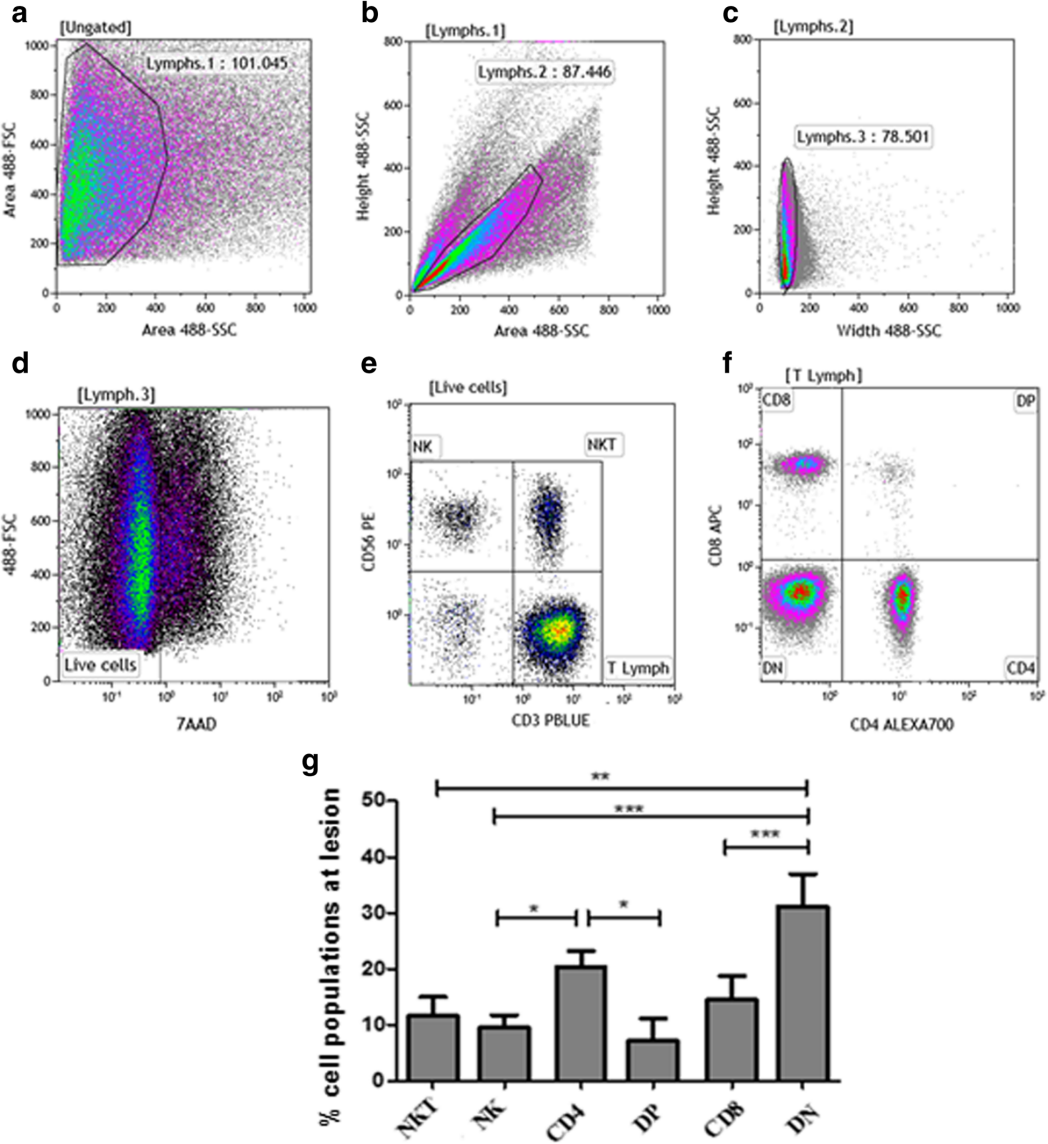
Frequencies of NK and NKT cells; CD4^+^, CD8^+^, double positive (DP) and double negative (DN) CD3^+^ T-lymphocyte subpopulations. **a**-**f** Flow cytometry-representative protocol: Cells obtained from cutaneous leishmaniasis lesions were stained with anti-CD3, anti-CD4, anti-CD8, anti-CD56, anti-CD107a and 7-AAD. Lymphocytes region was created on forward (FSC) *vs* side (SSC) scatter dot-plot (**a**). Cells gated on [Lymphs.1] were analyzed through *dot-plots* SSC-Height *vs* SSC-Area (**b**) and SSC-Height *vs* SSC-Width (**c**) to exclude doublets and debris. **d** Dead cells (7-AAD^+^) were excluded from the analysis and a gate encompassing 7-AAD^neg^ cells was performed. **e** Based on this gate, NK cells (CD56^+^CD3^neg^), NKT (CD56^+^CD3^+^) and T lymphocytes (CD56^neg^CD3^+^) were identified. **f** Based on T lymphocyte gate (T Lymph), CD4, CD8, double-negative (DN) and double-positive (DP) T lymphocytes were determined. **g** Bar graphs representing the mean ± SEM of percentages of NKT and NK cells; CD4^+^, DP, CD8^+^ and DN T lymphocytes obtained from cutaneous leishmaniasis lesions of 18 patients. Statistical analyses were performed using ANOVA test and Dunn’s *post-hoc* test. Results were considered significant with *P* < 0.05 (*P < 0.05; **P < 0.01; ***P < 0.001)

After the acquisition, this staining panel and gate strategies allowed us to evaluate the distributions of DN; DP T lymphocytes; NK cells; NKT cells; and cytotoxic-related CD107a expression, through a 7-parameter flow cytometry protocol using Kaluza analysis 1.5A software (Beckman Coulter) (Fig. 1a-f). The limits for the regions in dot plots and histograms were set based on non-staining cells, isotype controls and FMO control for CD107a.

### Cytometric Bead Array (CBA)

The cytokines measured were TNF, IL-8, IL-10 and IFNγ. The Cytometric Bead Array (CBA) Human Flex Set system (BD Biosciences) provides a method of capturing a soluble analyte or set of analytes with beads of a known size and fluorescence, enabling the detection of analytes using flow cytometry. The detection reagent supplied in the kit is a mixture of phycoerythrin (PE)-conjugated antibodies, which emits a fluorescent signal proportionally to the amount of bound analyte. When the capture beads and detectors reagents are incubated with an unknown sample containing identified analytes, sandwich complexes are formed. Granzyme B, IFN-γ and TNF-α levels were quantified in culture media from macerated lesions from ten patients. Thirty microliters of all samples were prepared following CBA multiplex kit manual, and the cytokines were detected within a range of 10–2,500 pg/ml. The assays were performed according to the manufacturer’s instructions, and samples were acquired in an FACSCallibur flow cytometer (BD Biosciences). The data were analysed with FCAP Array Software version 1-01 (Soft Flow, Inc., St. Louis Park, MN, USA).

### Statistical analysis

For statistical analyses between two groups at a time, we applied Mann-Whitney *U* test. For statistical analyses among more than two groups, we used one-way ANOVA test and Dunn’s posttest. We also used a Spearman’s rank correlation test. Correlations and intergroup differences were statistically significant when *P <* 0.05. We used GraphPad Prism 5.0 software (GraphPad Software Inc., La Jolla, CA, USA) for all statistical calculations and graphical representations.

## Results

### Frequencies of cell subsets in lesions of cutaneous leishmaniasis patients

We focused on determining the cytotoxic profile of cellular populations obtained directly from active cutaneous leishmaniasis lesions. A flow cytometry approach allowed us to evaluate the frequency of six populations: CD8^+^ and CD4^+^ T lymphocytes; CD4^neg^CD8^neg^ double-negative T lymphocytes (DN); CD4^+^CD8^+^ double-positive (DP) T lymphocytes; NK cells; and NKT cells (Fig. 1), as well as cells expressing cytotoxic-related CD107a biomarker. Our gating strategy was performed as follow: a Forward Scatter (FSC) *vs* Side Scatter (SSC) region was created using a density plot (Fig. 1a) to encompass the lymphocyte population. We also created height, area and width side-scatter dot plots, to remove doublets and debris from analysis (Fig. 1b, c). In addition, we included only live cells in analysis considering the gate comprising 7-AAD^neg^ (Fig. 1d) cells. Then, we defined the frequencies of T lymphocytes, NKT and NK cells based on CD56 *vs* CD3 dot plot (Fig. 1e). CD4^+^, CD8^+^, DN and DP cells were determined using a CD3^+^ T cell gate (Fig. 1f).

Regarding T lymphocyte populations, we observed that DN T lymphocytes showed the highest frequency (31.4 ± 4.6%), followed by CD4^+^ (21.4 ± 2.8%), CD8^+^ T lymphocytes (14 ± 3.3%) and DP T cells (7.0 ± 2.5%). NKT cells represented 11.5 ± 3.4% of total cell population and NK cells 9.4 ± 2.3%. The frequency of DN T cells was significantly higher than NK (*U* = 25.00, *P* < 0.0001), NKT (*U* = 42.00, *P* = 0.0003) and DP T cells (*U* = 50.00, *P* = 0.0007), while the frequency of CD4^+^ T lymphocytes was higher than NK (*U* = 45.00, *P* = 0.0004) and DP T cells (*U* = 66.00, *P* = 0.0043) (Fig. 1g).

### Frequencies of cell subsets expressing CD107a from lesions of cutaneous leishmaniasis patients

**Fig. 2:**
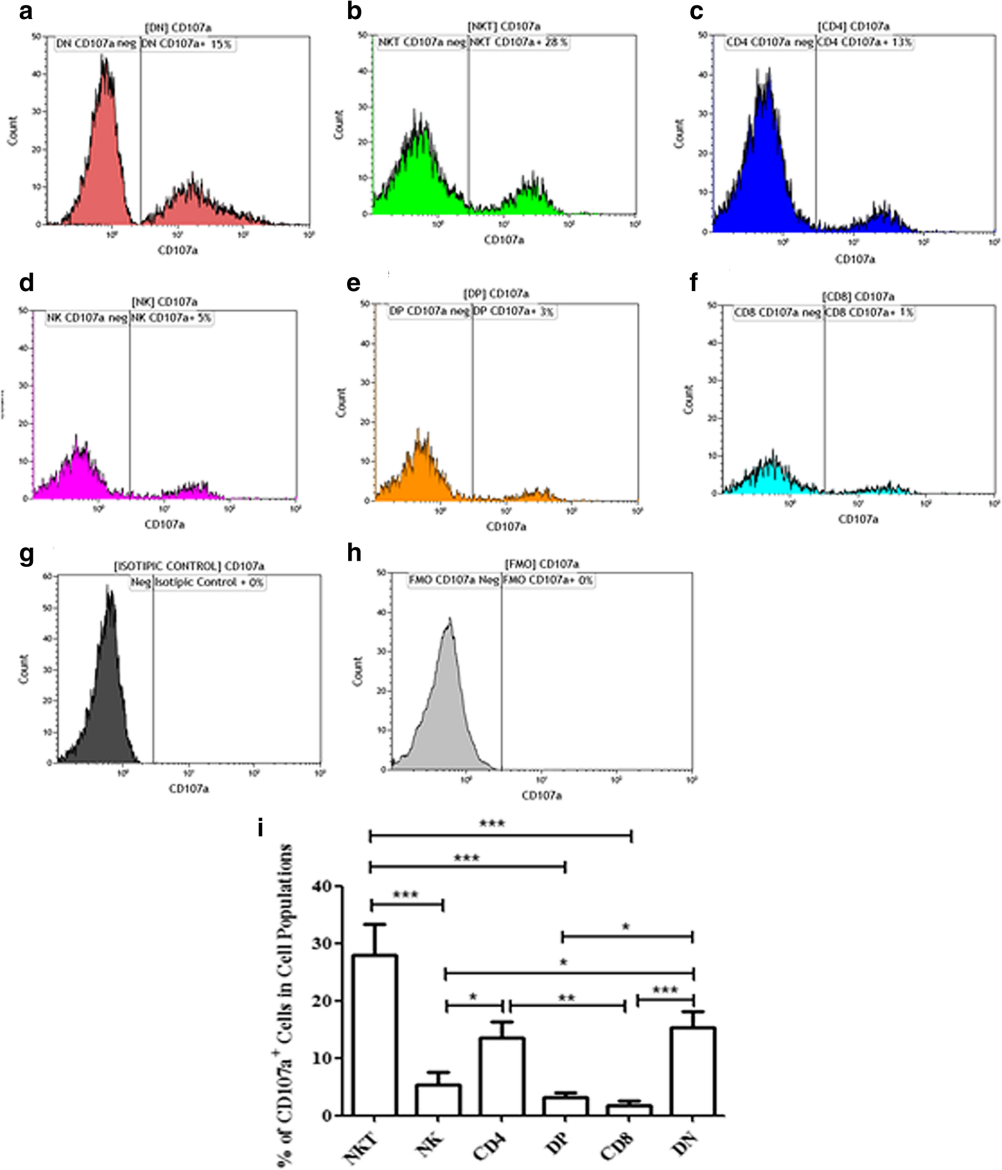
Frequencies of CD8^+^; CD4^+;^ DP; DN lymphocytes; NK and NKT cells expressing CD107a from lesions of cutaneous leishmaniasis patients. Flow cytometry-representative analysis: **a** CD107a^+^DN T; **b** CD107a^+^NKT; **c** CD107a^+^CD4^+^ T; **d** CD107a^+^NK; **e** CD107a^+^DP T; **f** CD107a^+^CD8^+^ T cells. Isotype control (**g**) and fluorescence minus one (FMO) for CD107a staining (**h**). Electronic gates were created surrounding NKT, NK, CD4, DP, CD8 and DN cell populations (see Fig. 1). **i** The bars graph representing the mean ± SEM of percentages of NKT and NK cells; CD4^+^, DP, CD8^+^ and DN T lymphocytes expressing CD107a obtained from cutaneous leishmaniasis lesions of 18 patients. Statistical analyses between two groups were performed using Mann Whitney non-parametric *t*-test. Results were considered significant with *P* < 0.05 (*P < 0.05; **P < 0.01; ***P < 0.001)

To assess the profile of CD107a^+^-degranulating-cell populations from the lesion site, we first evaluated their frequency within each cell-population studied. For this, we performed flow cytometric protocol to determine the frequencies of CD107a^+^ cells from gated total DN; NKT; CD4^+^; NK; DP; CD8^+^ cells (Fig. 2a-f, respectively). Figure 2i shows that CD107a^+^NKT cells (mean ± standard error: 27.9 ± 5.3%) displayed the highest frequency of positive cytotoxic cells within the pool of their population. In turn, CD107a^+^DN T (15.4 ± 2.8%) and CD107a^+^CD4^+^ T lymphocytes (13.5 ± 2.8%) showed similar frequencies to each other, but lower than that seen by CD107a^+^NKT cells (*U* = 114.0, *P* = 0.1329; *U* = 100.0, *P* = 0.05, respectively). CD107a^+^NK (5.2 ± 2.2%) showed lower frequencies in relation to NKT, CD4 and DN T cells (*U* = 40.0, *P =* 0.0001; *U* = 751.00, *P* = 0.0060; *U* = 61.00, *P =* 0.0014, respectively), as well as CD107a^+^DP T cells (3.04 ± 0.8%) (*U* = 17.50 *P* < 0.0001; *U* = 49.00, *P* = 0.0006; *U* = 42.5, *P* = 0.0003, respectively). Interestingly, CD107a^+^CD8^+^ T lymphocytes were found at the lowest frequency (1.8 ± 0.7%) and showed lower statistic difference when compared to NKT, DN and CD4^+^ T lymphocytes (*U* = 16.00, *P* < 0.0001; *U* = 27.00, *P* < 0.0001; *U* = 38.00, *P* < 0.0001, respectively) (Fig. 2i). It is important to note that based on this analysis we are not evaluating the total number of cytotoxic cells in lesion environment, but the percentages of CD107a^+^-degranulating cells considering the pool of each cell population as 100%.

### Contribution of cell subsets from the overall pool of CD107a^+^ cells

**Fig. 3:**
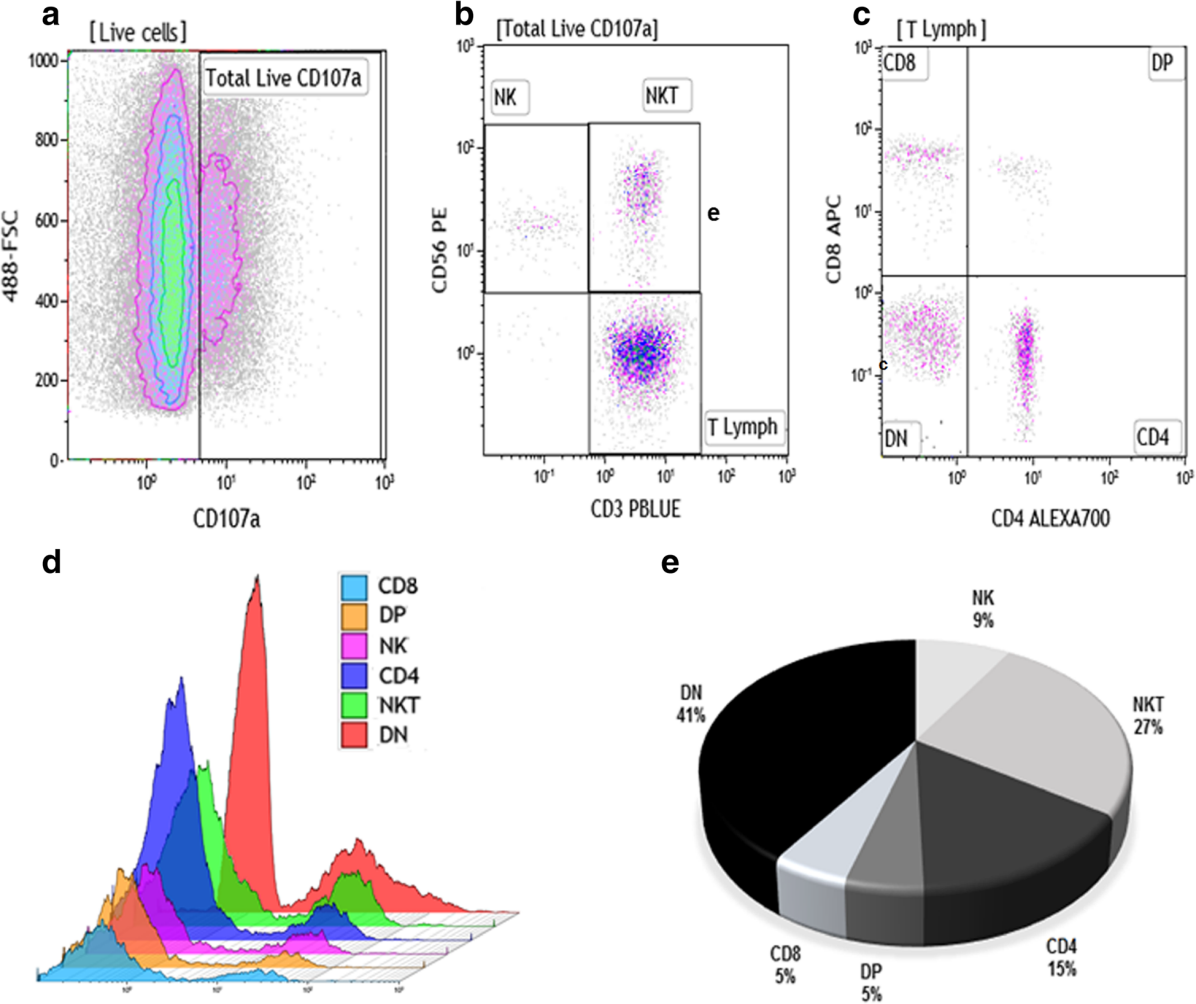
Distribution of CD8^+^; CD4^+;^ DP; DN lymphocytes; NK and NKT cells evaluated from the pool of CD107a^+^ cells. Flow cytometry-representative analysis to determine the distribution of cell populations in pool of CD107a^+^ cells. **a** FSC *vs* CD107a density-plot represents total percentage of CD107a^+^ cells based on [Live cells] gate (Fig. 1d). **b** CD3 *vs* CD56 density-plot gated on CD107a^+^ cells to define NK, NKT and T cells. **c** CD4 *vs* CD8 gated on CD3^+^ lymphocytes to define CD4, CD8, DP and DN T cells. **d** Overlay of CD8^+^; CD4^+^; DP; DN lymphocytes; NK and NKT cell histograms gated on CD107a^+^ cells. **e** Pie graph showing the mean data of NK and NKT cells; CD4^+^; DP, CD8^+^ and DN T lymphocytes (*n* = 18 CL lesions)

To determine the contribution of specific cell populations to the overall pool of CD107a^+^-degranulating cells in lesion environment, we also performed another flow cytometry approach, gating all CD107a^+^ cells (Fig. 3a), and then determine the percentages of each cell population studied from 100%-gated CD107a^+^ cells (Fig. 3b c). Thus, we observed that DN T cells were the major contributing cell population to the CD107a^+^-cell pool (40 ± 4%). NKT cells represented the second most prevalent population (25.0 ± 4.1%), followed by CD4^+^ T lymphocytes (14 ± 3.1%) and NK cells (8 ± 4%). DP and CD8^+^ T lymphocytes showed the lowest percentages inside the CD107a^+^-cell pool (4.0 ± 1.6 and 4.0 ± 2.3%, respectively) (Fig. 3d). In this context, comparing the distributions among the six different cell populations, based on the pool of CD107a^+^-degranulating cells, we observed that DN T cells were the major contributors to cytotoxic/degranulating cells at the lesion site, while the DP and CD8^+^ T lymphocytes were the lowest contributors.

### Correlation analysis of frequencies of CD107a^+^ cells with production of granzyme B, IFN-γ, TNF and clinical features

**Fig. 4:**
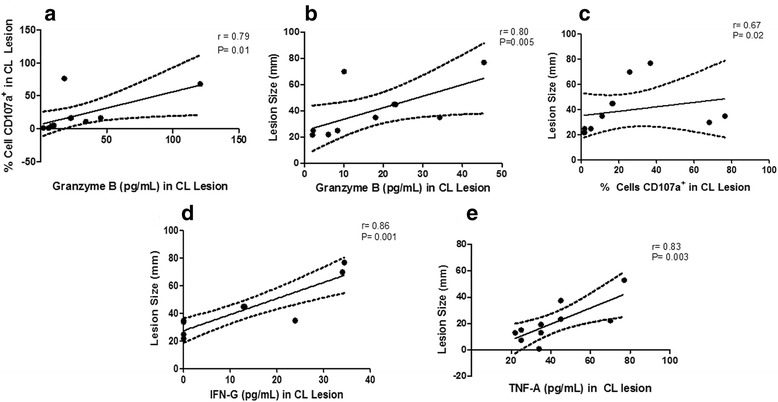
Correlation analysis of granzyme B, IFN-γ and TNF-α production, frequency of CD107^+^ cells and lesion size from lesions of CL patients (*n* = 10). Granzyme B, IFN-γ and TNF-α levels were quantified in culture media from macerated lesions biopsied from ten patients. All samples were prepared following CBA multiplex kit manual and the cytokines were detected within a range of 10–2500 pg/ml. We observed positive correlations between frequency of CD107a^+^ cells and granzyme B production (**a**); lesion size and granzyme B production (**b**); lesion size and frequency of CD107a^+^ cells in CL lesions (**c**); lesion size and IFN-γ production (**d**); and lesion size and TNF-α production (**e**). The central line represents median values and the graph show best fitted lines with 95% confidence interval. Statistical analyses were performed using Spearman’s test (r: correlation coefficient). Results were considered significant with *P* < 0.05. Each point represents one CL patient

To ascertain if there was a relationship between cytotoxicity/degranulation and clinical features, correlation analyses were carried out with the total frequency of cells expressing CD107a, granzyme B production and lesion size. Furthermore, we evaluated a possible interconnection between pro-inflammatory cytokines (IFN-γ and TNF-α - evaluated in culture media after the macerated-lesion process) with lesion size. We observed a positive correlation between the frequency of cytotoxic-CD107a^+^ cells and the production of granzyme B at the lesion site (*r* = 0.79; *P* = 0.01) (Fig. 4a). We also demonstrated that lesion size was positively correlated with; granzyme B production (*r* = 0.80; *P* = 0.005) (Fig. 4b); the frequency of CD107a^+^ cells (*r* = 0.67; *P* = 0.02) (Fig. 4c); IFN-γ levels (*r* = 0.86; *P* = 0.001) and TNF-α levels (*r* = 0.83; *P* = 0.003) (Fig. 4d, e, respectively). The other T cell subpopulations did not show any correlation with lesion size.

## Discussion

Lymphocytes are predominant mononuclear cell populations in leishmaniasis lesions and have been a focus of studies regarding the immunopathogenesis of CL. The literature-described role of CD8^+^ T lymphocytes in protective and pathological responses is still controversial. Previous observations from our group have shown an increase in the number of CD8^+^ T lymphocytes in the inflammatory infiltrate, suggesting cell recruitment to the CL lesion environment and a commitment to this cell population to lesion resolution [6–12]. In contrast, other authors have associated CD8^+^ T lymphocytes with tissue damage in CL and ML [12–17]. In murine models, CD8^+^ T cells are important for pathogen control but also are implicated in dermal pathology [25]. Apart from CD8^+^ T lymphocytes, other cell populations, such as NK and NKT cells, DN, DP and CD4^+^ T lymphocytes exhibit cytotoxic functions and could influence disease progression or healing.

Previous in situ immunopathological studies using confocal or fluorescent microscopy techniques in leishmaniasis have improved our expertise with the description of the cellular composition of the skin inflammatory infiltrate [18, 23, 24, 26, 27]. Flow cytometry (FCM) has been extensively applied as a key method to address these issues, and the majority of the reports are based on peripheral-blood-sample analysis [6, 11, 28–30]. However, it is known that lymphocytes are recruited from blood to lymph nodes, primed with antigens and then migrate to lesion sites; consequently, frequencies of antigen-specific T cells are higher in leishmaniasis lesions than in blood [7, 17, 31, 32]. For that reason, we assessed, using FCM, the cellular immune response in the CL-lesion environment, focusing on six distinct cytotoxic cell populations. Since tissue samples from CL lesions have a limited cellularity of lymphocytes compared to blood samples, interfering in the multigate-strategy FCM analysis, we standardised an enhanced protocol to obtain high-concentration-lymphocyte samples. Regarding another critical issue found in the literature, in which FCM protocols determine CD8^+^ T lymphocytes based only on CD8 expression, without NK and NKT exclusion, we added CD3 and CD56 to the analysis, allowing for exclusion of NK and NKT cells when analysing CD8^+^ or CD4^+^ T cells. Our data showed that the frequencies of CD4^+^ and CD8^+^ T lymphocytes are similar in the lesion-inflammatory infiltrate, though this similarity depends on the clinical profiles of the patients. Even with this resemblance, we observed that CD4^+^ and CD8^+^ T lymphocytes displayed different frequencies of CD107a^+^ cells, indicative of cytotoxic activity.

To investigate the participation of cytotoxic cell populations at the lesion site, we used two different FCM analysis approaches within the same sample. In one, we analysed the frequencies of each cell population expressing CD107a (degranulating cells indicative of cytotoxic activity). In the other, we evaluated the distribution of each cell population based on all (100%) CD107a^+^ cells. Interpreting data obtained from these two analyses led us to suggest that the NKT-cell pool had the largest portion of their cells dedicated to cytotoxicity, while DN T lymphocytes represent the most prevalent cell population looking to all CD107a^+^, degranulating cells. DP T cells with regulatory functions were observed in normal tissue and multiple sclerosis skin lesion [33, 34]. However, the recruitment of these cells to the skin is not fully elucidated, and in the periphery, these cells seem to exist as a mature population. It has been suggested that these cells originate from a CD8^+^ T cell precursor and then further express CD4 [35, 36]. Herein we noticed that this cell population is sparse in the CL lesion and their presence suggests their participation in the local immune response, and thus their recruitment to an inflammatory microenvironment. Furthermore, we showed that these cells are present in CL lesion with few participating as cytotoxic cells.

We also observed an overall low frequency of NK cells as compared to frequencies of CD4^+^ and DN T lymphocytes. This feature was already demonstrated in ML lesions were higher frequencies of NK cells in relapse cases in comparison to cured ones, suggested that high frequencies of NK cells could be a suitable protection/preventive prognostic marker [37]. Some studies have investigated the behaviour of NK and CD8^+^ T lymphocytes in CL, assuming that these cells are the main cytotoxic populations. The role of NK cells in CL has been associated with both pathology and protection. Some authors propose a protective function through lysis of extracellular promastigotes and infected macrophages [37, 38]. Nevertheless, there is evidence that cytotoxic NK cells contribute to exacerbation of tissue damage [39]. According to our investigations, only 5% of NK cells express CD107a, demonstrating a weak commitment of NK cell population to cytotoxicity and we observed a low frequency of cytotoxic NK cell in lesions (8% of all CD107a^+^-cytotoxic cells). These findings suggested that these cells have little influence on the cytotoxicity that occurs in the lesion environment, based on the distribution of total cytotoxic cells.

Regarding NKT cells, some reports showed that at least in CL murine models, these cells seem to block parasite expansion and also drive the immune response based on the cytokines produced [40, 41]. In humans, NKT cells play a role in several situations, for instance: showing either protective or pathogenic role against malaria; preventing autoimmunity; protecting against neoplasia, and having a direct pathogenic role against many opportunistic infections common in end-stage AIDS [42, 43]. In visceral leishmaniasis, NKT cells seem to have a dual behaviour, depending on their subset: CD4^+^NKT cells show a pathogenic activity and tend to accumulate at the infection site, while CD8^+^NKT cells may be protective when in contact with the target cells [44]. Our group has recently portrayed strong evidence about the involvement of CD8^+^ T and CD4^+^ T lymphocytes, NK and NKT cells (and their subsets) in the cytotoxic response analysing peripheral blood from CL patients before, during and after antimonial therapy. We reported an involvement of different NKT subsets in CL immunopathogenesis, showing CD8^+^NKT cells as the main subset involved in cytotoxicity and suggesting a protective role of DP NKT subset in CL [45]. There is no published report concerning the distribution of NKT cells in CL lesions. Results observed in our current study revealed that NKT cells were distributed as the fourth cell population found in CL lesions and they are the most committed to cytotoxicity, representing the second most cytotoxic-cell population in the CL lesion environment, pointing to them as an important component of the localised immune response.

Another important cell population observed in CL lesions is the CD4^neg^CD8^neg^ (double-negative - DN) T lymphocytes. DN T cells represent a minority subpopulation of mature post-thymic T lymphocytes that express CD3/TCRα/β or ɣ/δ receptor but lack CD4/CD8. These cells could play an inflammatory and regulatory role in immune response [46]. Other authors found that DN α/β T lymphocytes could be simultaneously both helper and cytotoxic activities [46–48]. Despite DN T cells representing a minority subpopulation in peripheral blood, this T cells subpopulation was also identified in the skin. Groh and cols. showed that CD3/TCR α/β complexes were functionally competent as evidenced by their capacity to transduce activation signals resulting in cell proliferation, cytokine secretion, and cytotoxic activity [49]. Here, we observed that, in addition to representing the greatest frequency of cells in the lesion (data not shown), DN T cells showed an important expression of the cytotoxic-related-CD107a^+^ phenotype.

In an experimental model, DN T cells seem to be key players in protective primary and secondary anti-*L. major* immunity [50]. In humans, some authors described the immunoregulatory potential of DN T cells in CL and reinforced their role in both protection and pathology. These cells exhibit a highly activated profile in active CL, being the second most prevalent producers of inflammatory cytokines, such as IFN-γ and TNF-α [46]. Furthermore, DN T cells could be subdivided into T cells expressing α/βTCR, which may be involved in an inflammatory response and T cells expressing γδTCR, which produces a biased regulatory environment [4, 46, 51]. We should consider that α/β DN T cells are associated with negative regulatory nature as well as with several autoimmune disorders. These DN subpopulations are restricted to CD1 presenting antigens, despite some cells express a restricted TCR and often recognise lipid antigens [46]. Given DN T cells are the predominant cytotoxic cells observed in CL lesions, we may hypothesise that, if the cytotoxicity mediated mechanisms lead to tissue injury, this might be due to a nonspecific lysis orchestrated by DN T cells.

Even with the high amount of DN cytotoxic cells in the lesion site, a great number of them are non-cytotoxic DN cells. For this reason, it is possible that DN T cells play a dual role, one as cytokine producers - inflammatory or regulatory - and another as a cytotoxic population. Our analyses showed that cytotoxic DN cells account for over 40% of all cytotoxic cells, ten times greater than CD8^+^ T lymphocytes. Thus, we show, for the first time, that DN T lymphocytes are present in CL lesion as the most frequent lymphocyte population and the largest subset of cytotoxic cells, emphasising a participation in the CL lesion milieu, likely related to a cytotoxic activity.

Interestingly, CD8^+^ T lymphocytes exhibited the lowest frequency of cytotoxic cells in CL lesions and did not seem to have a major participation in local cytotoxicity. Faria et al. [27] suggested that the frequency of CD8^+^ T lymphocytes expressing granzyme B was directly associated with the intensity of the inflammatory reaction of ulcerated lesions of CL. Other authors [13] described a lymphocyte recruitment and persistence of memory CD8^+^ T cells to the injury caused by *L. braziliensis*. It is important to remark that using flow cytometry; some authors did not evaluate CD8^+^ T lymphocytes gated on CD3^+^ and CD56^neg^ populations, which could allow the inclusion of other lymphocyte populations in the analysis, such as NK, NKT and gamma/delta + CD8+ T cells. Besides, NK cell expression of CD8 is significantly lower than CD8 expression by T cells and should be concerned by authors to gate on CD8 T cells (high-CD8 gating strategy). These procedures could avoid a misinterpretation of the flow cytometric data regarding the frequency of CD8^+^ T lymphocyte in CL lesions.

The role of CD4^+^ T lymphocytes in CL is well established as driving the immune response based on antigen presentation and cytokine profiles; nevertheless, few authors have concentrated efforts on studying cytotoxic functions of this lymphocyte subset. Recently, our group observed in peripheral blood, that the cytotoxic CD107a^+^CD4^+^ T cell might be involved in the healing process of CL patients [45]. Corroborating these findings, we observed in the current study a significantly high frequency of CD107a^+^CD4^+^ T-cell lymphocytes in the CL lesion milieu, surprisingly higher than classical-cytotoxic NK and CD8^+^ T cells. Others suggested CD4^+^ T cell-mediated cytotoxicity as a mechanism that assists viral control [52] and induction by *Trypanosoma cruzi* infection [53]. Thus, we may hypothesise that cytotoxic CD4^+^ T cells could take part in the immune response and contribute to parasite control in CL lesion through cytotoxic-related mechanisms.

It is important to note that a positive correlation among frequencies of CD107a^+^ cells, granzyme B production and lesion size provides evidence that cytotoxicity could be associated with tissue damage. In addition to cytotoxicity, tissue damage seems to be related to other pro-inflammatory factors, given larger lesion sizes are strictly associated with higher IFN-γ and TNF-α. Similar observations were seen by others, in which larger lesions were also correlated with a higher frequency of *Leishmania*-antigen-specific-inflammatory-cytokine (IFN-γ or TNF-α)-producing lymphocytes [54]

In summary, although several reports emphasise the key role of cytotoxic CD8^+^ T lymphocytes in CL tissue damage, we are unable to reinforce this hypothesis. This is reflected by the finding that CD8^+^ T cells represent the population with the least commitment to cytotoxicity (the lowest percentages of cytotoxic-related CD107a^+^ cells) in the lesions. Furthermore, CD107a^+^ cell pool. Nevertheless, based on our results, we are not able to affirm that CD8^+^ T lymphocytes do not have an immunomodulatory role, but we propose that cytotoxicity-mediated tissue damage observed in CL lesions seems to be more influenced by CD4^+^ T lymphocytes, NKT cells and mostly DN T lymphocytes.

## Conclusions

We focused our investigation on cytotoxicity-mediated mechanisms, which would occur in cutaneous lesions of CL patients and we showed, for the first time, the distribution and commitment of six distinct cytotoxic populations at the lesion site. From our findings, we suggest that cytotoxicity could have an important participation in the tissue damage observed in the lesion. However, we did not find evidence that CD8^+^ T cells are the main population responsible for this damage. Moreover, we may postulate that the major sources of cytotoxic activity are DN T lymphocytes, NKT cells and CD4^+^ T lymphocytes, which comprise 80% of all cytotoxic cells, although cytotoxic NK, DP and CD8^+^ T cell are detected in these lesions. These findings encourage us to look at cytotoxicity as a phenomenon that should be better explored, not only by classical-cytotoxic NK and CD8^+^ T cells but also by cytotoxic DN and NKT cells. Due to controversial statements regarding the actual role of these cell populations in the cytotoxicity and the complexity of the interaction between the human host and *Leishmania*, the study of the immunopathological mechanisms in humans naturally infected by *Leishmania* is of utmost importance, if we hope to develop effective vaccines and alternative immunotherapeutic treatments in the near future.

## Abbreviations

ATL: American tegumentary leishmaniasis
CBA: Cytometric bead array
CL: Cutaneous leishmaniasis
DN: Double negative
DP: Double positive
FCM: Flow cytometry
LAMP: Lysosomal-associated membrane protein
